# Inherited DNA-repair gene mutations in African American men with prostate cancer

**DOI:** 10.18632/oncotarget.27456

**Published:** 2020-01-28

**Authors:** Oliver Sartor, Shan Yang, Elisa Ledet, Marcus Moses, Piper Nicolosi

**Affiliations:** ^1^Tulane Cancer Center, Tulane University of School of Medicine, New Orleans, Louisiana, United States; ^2^Invitae Corporation, Genetics, San Francisco, California, United States

**Keywords:** prostate cancer, DNA repair, BRCA, African American, ethnicity

## Abstract

African American men with prostate cancer are understudied relative to Caucasians with prostate cancer with regard to testing for pathogenic germline DNA repair gene mutations. Herein we evaluate these two populations in a large commercial dataset and compare the detection of pathogenic/likely pathogenic alterations in 14 well annotated DNA repair genes (BRCA2, BRCA1, PALB2, ATM, RAD51C, CHEK2, PMS2, BARD1, BRIP1, MLH1, MSH2, MSH6, NBN, and RAD51D). Overall, pathogenic or likely pathogenic alterations in these 14 DNA repair genes were less likely to be detected in African Americans as compared to Caucasians. Upon a more in-depth analysis, the risk of germline pathogenic/likely pathogenic BRCA mutations was similar between the two populations whereas there was a lower risk among African Americans for the non-BRCA mutations. No African American men were noted to have mutations in BARD1, BRIP1, MLH1, MSH2, MSH6, NBN, and RAD51D in this data set. Stage, grade, and metastatic status were not assessed in this group of patients. Larger and more detailed studies conducted in men with prostate cancer are required to confirm these findings.

## INTRODUCTION

African American (AA) men are incompletely characterized with regard to germline DNA repair mutations in the prostate cancer data sets published to date. In general, AA men with prostate cancer are under-represented in clinical trials and genetic studies. Herein we use a large commercial DNA germline assessment data set to compare frequencies of pathogenic/likely pathogenic (P/LP) alterations in 14 well characterized DNA repair genes assessed in both AA men and Caucasian American (CA) men with prostate cancer.

## RESULTS

The frequency of pathogenic or likely pathogenic (P/LP) germline variants in 14 genes involved with DNA repair (see Table 1) were compared between AA and CA with prostate cancer using Invitae panel-assay assessed patients. This commercial laboratory has samples derived from a broad representation of private and academic practices based in the United States. These particular 14 genes are well curated germline genes previously published on by Pritchard et al. [1].

**Table 1:** Details on gene assays and comparison to Pritchard et al. [1] and an Invitae data set for men with prostate cancer

Gene	Invitae AA Men^*^ tested	% P/LP	Invitae CA^**^ men tested	% P/LP	Invitae AA vs CA P value	Pritchard [1] tested	% P/LP Pritchard [1]
*ATM*	206	0.97%	2302	2.09%	0.27	692	1.59%
*BARD1*	140	0.00%	1695	0.00%	—	561	0.00%
*BRCA1*	213	1.41%	2469	1.17%	0.76	692	0.87%
*BRCA2*	214	2.80%	2488	4.78%	0.18	692	5.35%
*BRIP1*	148	0.00%	1779	0.39%	—	561	0.18%
*CHEK2*	207	0.48%	2379	3.11%	0.03	534	1.87%
*MLH1*	212	0.00%	2402	0.08%	—	692	0.00%
*MSH2*	212	0.00%	2408	0.79%	—	692	0.14%
*MSH6*	213	0.00%	2403	0.37%	—	692	0.14%
*NBN*	200	0.00%	2262	0.44%	—	692	0.29%
*PALB2*	182	1.10%	2171	0.60%	0.42	692	0.43%
*PMS2*	212	0.47%	2403	0.42%	0.90	692	0.29%
*RAD51C*	148	0.68%	1760	0.23%	0.31	692	0.14%
*RAD51D*	162	0.00%	1940	0.15%	0.61	692	0.43%

^*^AA = African American, ^**^CA = Caucasian American.

In these canonical 14 genes, in our germline data set, there were a total of 16/214 (7.5%) AA men and 347/2488 (13.9%) CA men with pathogenic germline findings (*p* = 0.008 by Chi square testing). As shown in Table 1, the P/LP variants in AA men were in *BRCA2* (6/214, 2.8%), *BRCA1* (3/213, 1.4%), *PALB2* (2/182, 1.1%), *ATM* (2/206, 1.0%), *RAD51C* (1/148, 0.68%), *CHEK2* (1/207, 0.48%), and *PMS2* (1/212, 0.47%). No AA men in this data set had P/LP mutations in *BARD*1, *BRIP1*, *MLH1*, *MSH2*, *MSH6*, *NBN*, or *RAD51D*.

It is notable that the 2 *BRCA* genes frequency of P/LP alterations was not distinct between AA and CA men (*p* = 0.30). However, the 12 non-*BRCA* genes in this panel occur at a lower frequency among AA men as compared to CA men. A total of 7/2242 (0.31%) individual gene tests in AA men had non-*BRCA* P/LP alterations as compared to 199/25904 (0.77%) individual gene tests in CA men. These differences were significantly different when assessed by Chi-Square (*p* = 0.015).

When comparing individual genes in the AA and CA cohorts, *CHEK2* was less frequent among the Invitae-derived AA men as compared to Invitae-derived CA men (0.48% vs 3.11%, *P* = 0.03); other genes were not distinct. In the CA population, 36.8% of the *CHEK2* mutations are 1100delC. None in the AA population have that mutation.

Importantly, comparing the overall frequency of P/LP variants of these same genes in CA men assayed in the current cohort with that of the predominantly CA data set from Pritchard et al. [1] revealed no differences in P/LP variants (13.9% here vs 11.8% in Pritchard et al., *P* = 0.15).

## DISCUSSION

AA men with prostate cancer are less likely to have P/LP among the 14 assayed DNA repair genes compared to CA men in our cohorts. The 12 non-*BRCA* P/LP alterations as a whole were clearly less frequent in AA men. CHEK2 P/LP alterations are specifically less commonly encountered in AA men. Though our data suggest that non-BRCA germline DNA repair mutations are lower in AA men, these data need verification in larger and better annotated datasets. CHEK2 is of considerable importance in explaining the lower prevalence of non-BRCA mutations in these AA men.

Lack of stage, Gleason scores, and family history are notable in this analysis using the Invitae dataset and represent a limitation of this study, but presumptively (though not verifiably) the biases equally apply to both the AA and CA subsets. Unknown biases may confound the interpretation of these data.

AA men are less likely to have localized prostate cancer at diagnosis, and more likely to have local or regional spread [2]. In fact these authors are unaware of any comparative data set that suggests that AA men are diagnosed with less advanced disease than CA in the United States. This is important as it makes it unlikely that the lower rates of AA P/LP variants detected herein are attributable to less advanced disease.

The similarity between our CA cohort and that of Pritchard et al. [1] study indicate that our Invitae-derived prostate cancer patient populations are not significantly distinct as compared to a well cited predominately CA data set, thus signifying the generalizability of our Invitae-derived CA population; however, the lack of stage, Gleason, and family history make potential biases in the AA and CA subsets possible but unverifiable. Of note the AA population in the Pritchard study was 5.8% [1].

Though these data in AA are relatively small, they are the largest reported to date. These data indicate that the excess risk of prostate cancer in AA men [3] cannot be explained by an excessive number of DNA defects in these 14 canonical genes. Somatic mutations are not addressed herein but represent an important issue in assessments for precision therapies [4, 5]. We recognize that not all of these mutations, such as the CHEK2 mutation, may be actionable at this time but these data may have implications as precision therapeutics evolve.

## MATERIALS AND METHODS

Germline testing for DNA repair defects was performed by a certified lab, Invitae (Invitae.com, San Francisco, California, USA), in men with prostate cancer. Physicians ordered this testing in the real world setting and details on age of cancer onset, stage, and family history are not reliably available. Thus, we utilized comparable data sets derived from Invitae testing, in AA men and CA men. Classification of race was done by assessment of categories specified in the intake form filled out by physician offices and submitted to the central lab. Separately we utilized a highly regarded germline detailed data set [1] to assess the comparability of the Invitae data to an independent dataset. Identical panels were not used in all men, both in our assays and that of Pritchard et al. [1], thus variations in the number of assays analyzed are noted from gene to gene.

Chi square testing was used to compare proportions. No *P* values are calculated when the number of P/LP cases in the AA subset equaled zero, given limitations of Chi-Square testing when the numerator is zero.
